# Report of the 23rd Meeting on Signal Transduction 2019—Trends in Cancer and Infection

**DOI:** 10.3390/ijms21082728

**Published:** 2020-04-15

**Authors:** Bastian Schirmer, Klaudia Giehl, Katharina F. Kubatzky

**Affiliations:** 1 Institute of Pharmacology, Hannover Medical School, 30625 Hannover, Germany; 2 Signal Transduction of Cellular Motility, Internal Medicine V, Justus Liebig University Giessen, 35392 Giessen, Germany; Klaudia.Giehl@innere.med.uni-giessen.de; 3 Department of Medical Microbiology and Hygiene, Heidelberg University Hospital, 69120 Heidelberg, Germany; kubatzky@uni-heidelberg.de

**Keywords:** signal transduction, STS, conference report, receptor signalling, neuronal signalling, infection and inflammation, growth factors, cytokines, immune cell signalling, cancer research, tumour biology

## Abstract

The annual meeting “Signal Transduction–Receptors, Mediators and Genes” of the Signal Transduction Society (STS) is an interdisciplinary conference open to all scientists sharing the common interest in elucidating the signalling pathways underlying the physiological or pathological processes in health and disease of humans, animals, plants, fungi, prokaryotes and protists. The 23rd meeting on signal transduction was held from 4–6 November 2019 in Weimar, Germany, and focused on “Trends in Cancer and Infection”. As usual, keynote presentations by invited scientists introduced the respective workshops and were followed by speakers chosen from the submitted abstracts. Ample time had been reserved for discussion of the presented data during the workshops. In this report, we provide a concise summary of the various workshops and further aspects of the scientific program.

## 1. Introduction

The 23rd Meeting on “Signal Transduction—Receptors, Mediators and Genes” was held in Weimar, Germany, from 4 to 6 November 2019 [1]. This year, the meeting was organized by the Signal Transduction Society (STS) together with the signalling study groups of the German Societies for Pharmacology (DGP), Cell Biology (DGZ), Biochemistry and Molecular Biology (GBM), and Immunology (DGfI). The collaborative research centre (SFB) 854 “Molecular Organization of Cellular Communication in the Immune System” (B. Schraven, Magdeburg, DE), the study group “Infection Immunology” of the DGfI and the German Society for Hygiene and Microbiology (DGHM), as well as the study group B cells of the DGfI provided further scientific and financial support. In keeping with the focus on “Trends in Cancer and Infection” a special workshop had been organized together with the German Centre for Infection Research (DZIF). The number of different organizations involved highlights the interdisciplinary approach of the meeting that brings scientists together at the interphase of different research areas to stimulate fruitful discussions and collaborations. In order to integrate young scientific organizations, the Young Investigators of the German Society for Biochemistry and Molecular Biology served as co-organizers of a special workshop. Additionally, abundant time was reserved for young scientists to present their work as posters in the two very busy poster sessions or as oral presentations during the meeting.

## 2. Meeting Overview

### 2.1. Focus Theme Workshops

#### 2.1.1. Workshop on Infection and Cancer

This workshop had been organized in collaboration with the DZIF and was opened by two renowned experts in the field, namely Céu Figueiredo (Porto, Portugal) and Mathias Heikenwälder (Heidelberg, Germany).

Céu Figueiredo presented her work on the role of bacterial populations in gastric cancer to the audience. A significant percentage of gastric cancers can be attributed to infections with the pathogenic bacterium *Helicobacter pylori*, which is now classified as a class I carcinogen. Indeed, the presence of *H. pylori* virulence factors correlates with an increased transformation of gastric lesions into gastric cancer [2]. This chronic bacterial colonization leads to a persistent pro-inflammatory state, increased cell proliferation, cell survival and genetic instability of the gastric epithelium, which is the reason for the observed higher risk in gastric transformation [3]. Figueiredo’s group hypothesized that the composition of gastric microbiota modifies the *H. pylori*-induced gastric neoplasia. Indeed, gastric carcinogenesis was promoted by the presence of complex microbiota in a mouse model of gastric cancer [4]. However, patients suffering from gastric cancer showed a lower microbial diversity and a different microbiota composition as compared to patients suffering from chronic gastritis. Applying a standardized microbial dysbiosis index to assess the gastric microbiota composition, it was possible to discriminate between chronic gastritis and gastric cancer. Interestingly, especially those microbiota able to produce carcinogenic N-nitroso compounds were found enriched in gastric cancer, whereas the proportion of *H. pylori* in the microbiota decreased during gastric carcinogenesis [5].

The following keynote presentation by Mathias Heikenwälder (Heidelberg, Germany) switched focus to liver cancer, in which different types of immune cells are found to drive progression from non-alcoholic steatohepatitis (NASH) to cancer. Due to the high prevalence of obesity in the Western countries nowadays, NASH has become a relevant cause for liver cancer. A key finding in NASH is sterile inflammation, which is aggravated by both the innate and the adaptive immune system [6]. Heikenwälder’s group developed several mouse models to study the progression from obesity-associated NASH to hepatocellular carcinoma (HCC) and found that a choline-deficient high fat diet was able to mimic the progression from metabolic syndrome over NASH to HCC in mice. In the livers of these mice, cytotoxic T cells and NKT cells were found to be activated and to promote HCC development. Of note, the presence of activated NKT cells led to the activation of lymphotoxin (LT)βR-signalling in hepatocytes, eventually causing steatosis. Additionally, both CD8^+^ T and NKT cells caused increased NF-κB signalling in hepatocytes and, thus, promoted the progression of NASH to HCC [7]. In order to treat the described intrahepatic inflammation in NASH prior to HCC transformation, antiplatelet therapy can be an option, as Heikenwälder demonstrated during the remainder of the talk. Kupffer cells could be demonstrated as key players in platelet recruitment to the liver, and platelet glycoprotein Ib alpha chain (GPIbα) from platelets is involved in Kupffer cell binding during NASH progression. By releasing their α-granula contents, platelets might significantly contribute to the chronic intrahepatic inflammation. In agreement with this hypothesis, antiplatelet therapy was shown to prevent transformation of NASH into HCC and to partially revert NASH in a mouse model [8].

#### 2.1.2. Workshop on Tumour Biology and Immunity

Opening this workshop, Romina Goldszmid (Bethesda, Rockville, MD, USA) highlighted the importance of microbiota for regulation of the tumour microenvironment. During anticancer therapies the tumour microenvironment is changed and as a consequence, tumour-destructive immune responses are mounted. The role of the microbiota in modulating the immune response has long been unclear, but there is growing evidence that commensal bacteria alter local inflammation processes and affect therapy responses in cancer. Goldszmid was able to show that mice, which had received a subcutaneous injection of MC38 colon carcinoma cells, only responded well to an intra-tumoural immune therapy with anti-IL10R and the TLR9 ligand CpG when they had their “native” microbiome. After antibiotic combination therapy that drastically reduced the complexity of the microbiota, production of TNF-α by tumour myeloid cells was significantly impaired. Indeed, analysis of 16S ribosomal RNA gene copy numbers showed that the tumour TNF response depended on the composition of the commensal microbiota detected in the faeces of the mice. Another important clinical aspect of the microbiota was uncovered with mice bearing the T lymphocyte-derived EL4 tumour cell line. The success of a therapy with oxaliplatin clearly depended on the intact microbiome, which intratumourally increased ROS production, as mice treated with antibiotics exhibited a significantly lower survival rate after therapy [9]. Neutrophils are the first mediators of the innate immune defence, however, in cancer they are associated with poor prognosis although their role is not well-defined. Goldszmid explained that neutrophils in circulation and those contained within the tumour are different regarding their transcriptional phenotype and their ability to generate ROS. Interestingly, in the absence of microbiota less neutrophils are found in the tumour. In addition, trained immunity increases ROS production suggesting that the gut bacteria help fight the tumour [10].

Lionel Larue (Orsay, France) focused in his talk on skin cancer and the transition from melanoblasts to melanomas. His group investigates the establishment and the renewal of the melanocyte lineage, as well as melanomagenesis. Although therapy options improved over the last ten years, there is still a significant need for efficient treatment options. A possible reason for this could be that melanoblasts evolved very late and thus bear only limited similarity to other lineages. In his talk he concentrated on two proteins, namely MiTF and Brn2 during melanoma initiation and progression. Brn2 is a tissue-restricted POU domain transcription factor which plays a key role in promoting invasion and regulating proliferation The MiT/TFE transcription factor family which belongs to the MYC superfamily of basic helix-loop-helix leucine zipper (bHLH-ZIP) proteins. Melanoma tumours with activating β-catenin mutations exhibit enhanced MiTF and Brn2 expression. Larue shows that Mitf-M affects proliferation, survival and transformation of melanocytes by interfering with cyclin-dependent kinase 2 and 4, p21 and BRN2 [11,12].

#### 2.1.3. Workshop on Immune Cell Signalling and Cancer

The workshop was opened by the inspiring keynote presentation of Claudia Kemper (Bethesda, MD, USA), who shed light on the key role of the complosome for maintaining normal physiology. Whereas extracellular complement has long been known to sense and combat pathogens, the intracellular complement system (CD46, C3a, C3b) termed “complosome” has just recently entered scientific literature. Kemper and her group were able to show that the complosome participates in maintaining normal physiological conditions in numerous immune cells. For example, intracellularly-activated autocrine C3a and C3b could be demonstrated to support Th1 induction and intracellular C3a generation is an important tonic activator of mTOR to allow T cell survival. A compromised complosome function is associated with a substantial number of immune cell-driven diseases [13]. The intracellular CYT-1 fragment from CD46 can, after being cleaved off from the full-length protein, bind to several glycolytic enzymes including GAPDH. Binding of CYT-1 to GAPDH dissociates GAPDH multimers to mono-, di- and trimers, which can serve distinct “moonlighting” functions. In case of GAPDH, interaction with CYT-1 induces GAPDH-mediated increase in GLUT1 mRNA translation, so CD46 may serve as a switch for the function of key glycolytic enzymes (unpublished data). Furthermore, by impacting expression of many metabolic enzymes, the C3-CD46 axis is required for adequate IFN-γ production in Th1-polarized T cells and for optimal activity of cytotoxic T cells [14]. Via crosstalk with the NLRP3 inflammasome, both C3 and C5 are able to regulate T cell responses [15]. These novel, non-canonical activities of the complement system will enable a deeper understanding of T cell regulation in the future.

The ensuing keynote presentation by Thomas Oellerich (Frankfurt a. M., Germany), focused on the elucidation of oncogenic B cell receptor (BCR) signalling in diffuse large B cell lymphoma (DLBCL), more specifically in the subgroup of activated B cell-like (ABC) lymphoma. The Oellerich laboratory uses proteogenomics to identify new drug targets, uncover mechanisms of drug response, and identify predictive biomarkers. An interesting conundrum, which has been tackled by the work group, is the response of only certain ABC lymphoma cells to Bruton’s tyrosine kinase (BTK) inhibition by the BTK inhibitor ibrutinib. Using CRISPR/Cas9 screens, phosphoproteomics and functional/structural genomics, TLR9 could be identified as a vulnerable point in the BCR signalling network of CD79/MyD88-mutant cells. It could be further demonstrated that in ibrutinib-responsive ABC cells TLR9 directly interacts with the BCR in the endolysosomal compartment forming a “My-T-BCR complex” consisting of MyD88, TLR9, and the BCR. Since mTOR was found as another component of this My-T-BCR complex, the combined inhibition of mTOR and BTK showed indeed synergistic effects on ABC cell proliferation [16]. Altogether, the My-T-BCR complex is a pro-survival signalling hub, which is of high relevance for ibrutinib response and might be used as predictive biomarker in the future. Cancer gene resequencing of patients diagnosed with ABC lymphoma could then be performed to identify those patients with a high probability of response to ibrutinib as a step forward towards precision medicine.

### 2.2. Other Workshops

The workshop on *Cytokines, Growth Factors and Their Receptors* was opened by Anne Ridley from Bristol, UK, who introduced the central role of Rho GTPase networks in cell migration to the audience. Ridley’s group performed RNAi screens of various Rho GTPase signalling networks in PC3 prostate cancer cells in presence or absence of hepatocyte growth factor (HGF) and identified RhoH as an unexpected hit. Formerly, RhoH expression was described to be restricted to hematopoietic cells and absence of RhoH had been associated with a variety of B cell cancers [17]. Ridley could show that high expression levels of RhoH in patient-derived prostate cancer specimens correlated with a poorer prognosis. Depletion of RhoH in vitro led to a phenotype with decreased migratory polarity resembling constitutively active Rac1. Since RhoH could be shown to interact with both Rac1 itself and the shared effector p21-activated kinase (PAK) 2, RhoH may affect cancer cell migration and invasiveness by facilitating correct localization and function of Rac1 and PAK2 [18].

Stephan Ludwig (Münster, Germany) held the keynote lecture in the workshop on *Differentiation, Stress, and Death*. He presented his work on intracellular signal transduction cascades induced by influenza virus infections, focusing on the identification of cellular signalling molecules, which can be exploited as therapeutic targets. During influenza virus infection, viral hemagglutinin (HA) initiates MAP kinase signalling via the Raf/MEK/ERK/Rsk pathway [19,20]. Ludwig’s team was able to show that impairment of this HA/MAPK pathway by siRNA against ERK1/2 or using MEK inhibitor CI-1040 leads to decreased nuclear export of viral ribonucleoproteins (vRNP) by blocking Rsk-mediated phosphorylation of vRNP at S269 and S392. Since the MEK inhibitor CI-1040 blocked replication of several influenza virus strains and showed no indications of generating resistant virus variant, the Ludwig group decided to prove feasibility of antiviral therapy in a mouse model, where CI-1040 indeed led to prolonged survival [21,22]. Since the drug has already been shown to be well tolerated in humans (Phase I trial, EudraCT 2019-000784-25), MEK inhibitors are promising antiviral drugs against influenza.

The GBM Young Investigators had invited Viktor Korolchuk (Newcastle upon Tyne, UK), to open the workshop on *Signalling from Intracellular Organelles*. Being committed to ageing research, Korolchuk introduced the central role of mammalian target of rapamycin complex 1 (mTORC1) and the lysosomal compartment in cellular senescence. A key regulator of mTORC1 is the lysosome that serves as a physical platform allowing mTORC1 activation but also represents the ultimate compartment where autophagy takes place [23]. In senescent cells, mTORC1 may become constitutively active and “blind” to exogenous nutrient starvation. This is, on the one hand, due to increased flow through the autophagic pathway resulting in higher lysosomal amino acid content. On the other hand, a depolarization of the senescent cell membrane leads to primary cilia defects and a persistent activation of the PI3K/Akt pathway, which finally relieves inhibition of mTORC1 by the tuberous sclerosis complex (TSC) [24]. Interestingly, the lysosomal positioning within the cell also plays a major role in mTORC1 regulation, because the lysosome serves as a “platform” for mTORC1. Starvation, for example, leads to the clustering of lysosomes near the microtubule organizing centre and favours autophagic processes. After nutrient replenishment, lysosomes are scattered over the cell allowing bound mTORC1 to be activated by signalling molecules located in the cell periphery [25].

The workshop on *Tumour Biomarkers and Oncogenes* started with the keynote presentation of Carsten Denkert (Marburg, Germany), shining a light on the suitability of different molecular biomarkers for prediction of therapeutic responses in breast cancer. In 15% of breast cancer patients, the tumour turns out to be triple negative breast cancer (TNBC), i.e., it shows neither oestrogen or progesterone receptor positivity, nor HER2/neu expression. Tumours can be further classified according to the prevalence of tumour-infiltrating lymphocytes (TIL). In early TNBC, a high amount of TIL (>60%; “immunologically hot tumour”) is predictive of an increased chemotherapy response (neoadjuvant) and better survival [26,27]. Moreover, expression levels of immune genes—regardless if anti- or proinflammatory—were found to correlate with the chemotherapy response, so that a combination therapy with chemotherapeutics and immune checkpoint inhibitors is conceivable. Indeed, Denkert presented numerous data showing the potential synergism between classical chemotherapy, which generates cancer antigens, and immune checkpoint inhibition, which boosts the immune response to the antigens. Importantly, care must be taken to identify those patients profiting from a combination therapy. The immune checkpoint protein PD-L1, for example, may serve as a biomarker for immune therapy response in metastatic, but not early TNBC [26].

Jan Parys (Leuven, Belgium) opened the session on *Calcium Signalling* with his comprehensive presentation of the interrelation between oncogenes, the IP_3_ receptor and subsequent calcium signalling in cancer. One oncogene regulating IP_3_ receptor function is the anti-apoptotic protein Bcl-2, which is upregulated in several hematologic malignancies such as diffuse large B-cell carcinoma and chronic lymphocytic leukemia. As several oncogenes attenuate IP_3_R signalling and exaggerated calcium release leads to cell death, understanding the regulatory mechanisms of IP_3_R might lead to novel therapeutic approaches in cancer. Based upon the IP_3_R binding site in Bcl-2, Parys’ group developed the cell-penetrating peptide BIRD-2, which was able to efficiently block Bcl-2/IP_3_R interaction and triggered cell death in DLBCL and CLL cell lines, especially when IP_3_R expression was inherently high. However, surprisingly, Bcl-2 was able to inhibit calcium release from the ER even if the central regulatory Bcl-2 binding site had been deleted from IP_3_R [28]. This observation led to the discovery of two other binding sites for Bcl-2 in the IP_3_R. One is located within the C-terminus of the protein and is important for Bcl-2 recruitment to the IP_3_R and for mediating the anti-apoptotic effect of Bcl-2. Since the other one is located within the ligand binding domain of IP_3_R at the N-terminus, it came as no surprise that Bcl-2 could be displaced at this site by high concentrations of IP_3_R agonists. Indeed, high amounts of IP_3_ were able to overcome Bcl-2-mediated inhibition of IP_3_R in vitro [29,30,31].

### 2.3. Fostering of Early Career Researchers

In 2019, the STS grant committee chose 5 students to receive travel grants of 250 € each to allow their meeting attendance. Of these travel stipends, the two Silver Sponsors of the meeting, Jackson ImmunoResearch and Biomol, sponsored one grant each. As a follow-up of the well-known ‘one Minute-One Transparency’ session, each participant of the meeting had the chance to promote their research in a “My Poster in One Minute” flash talk to the audience, allowing the presenters to attract the listening scientists to their posters. Afterwards, everyone had the possibility to mingle with other scientists at the posters in a casual atmosphere, to discuss presented data, and to establish contacts. The chairpersons of the meeting had the opportunity to visit the posters and select five award winning posters out of the many contribution, which were awarded prizes to a total value of 750 €.

The STS Science Award is offered as a reward for excellent research by an early career researcher of the STS. It was first introduced in 2005 and has become a regular element of the annual STS Meetings. In 2019, the STS Science Award was awarded to Claudia Stäubert (Leipzig, Germany) for her innovative research on the role of hydroxycarboxylic acid receptor 3 (HCA_3_) as modulator of human immune function. Specifically, she could identify D-phenyllactic acid, which can be detected in food fermented by lactic acid bacteria (e.g., sauerkraut), as potent activator of HCA_3_ serving as a chemotactic receptor in human monocytes [32]. The award, with a total sum of 1500 €, was donated by the Signal Transduction Society.

## 3. The STS Honorary Medal Award

The STS Honorary Medal was introduced in 2010 in order to honour outstanding scientists in the field of signal transduction. Previous winners of the award are Anthony Pawson, Tony Hunter, Carl-Henrik Heldin, Klaus Rajewsky, Jules Hoffmann, Mina Bissell, Tak Wah Mak, Michael Reth and Karen Vousden. Since 2017, the medal is awarded by the STS in collaboration with the International Journal of Molecular Sciences (IJMS), a journal of the MDPI publishing group. In 2019, Alfred Wittinghofer (Dortmund, DE) received the STS Honorary Medal Award for his influential work on structure-function relationships of GTP-binding proteins and their involvement in physiological and pathophysiological processes. Wittinghofer resolved the 3D-structure of Ras and characterized its interaction with effector and regulator proteins on the molecular and structural level, which also led to the discovery, why oncogenic Ras proteins are constantly active [33,34]. His research revolutionized our view on GTPases and guided the development of novel therapeutic approaches targeting cancer [35,36]. The award ceremony was opened by a personal laudation for the awardee by Gudula Schmidt, a former student from the Wittinghofer lab and now a principle investigator at the University of Freiburg, and the festive presentation of the medal by the STS council. The medalist then gave the “Honorary Medal Lecture” where he presented an overview of his many years of research, but also introduced his more recent work. Here, Wittinghofer focused on innovative efforts to establish the connection between Ras signalling and ciliary trafficking. The GTPases Arl2 and Arl3, belonging to the Arf subfamily of Ras proteins, can act, when GTP-bound, as release factors for the carrier proteins PDE6δ and UNC119. These carrier proteins are essential for proper intracellular (and intraciliary) localization of several N-myristoylated (UNC119) or farnesylated (Arl) proteins [37,38,39]. As an example, Wittinghofer presented his innovative work leading to elucidation of the ciliary trafficking mechanism of farnesylated inositol phosphatase INPP5 [40]. The lecture was followed by a vivid discussion of the past and future perspectives regarding Ras (patho) physiology and drug development.

## 4. Final Remarks

The 24th STS Meeting will again take place at the Leonardo Hotel in Weimar from November 2nd to November 4th 2020. The organizers would like to encourage the participation of scientists from all fields related to signal transduction to strengthen the interdisciplinary character of this meeting. Especially we hope to link and share concepts on “Target identification and Intervention”—the focus topics of the 2020 meeting.

Preparations for the meeting have already started and regular updates on the schedule and contents of the meeting can be found at https://www.sigtrans.de. Additionally, news regarding the work of the STS can be accessed via the Facebook or through the Twitter account (@SignalSociety).
